# Laser Interstitial Thermal Therapy for High-Grade Gliomas: Current Evidence, Clinical Applications and Emerging Role of Artificial Intelligence

**DOI:** 10.3390/jcm15155928

**Published:** 2026-07-29

**Authors:** Sergey Chudievich, Maria Pospelova, Alexey Ulitin, Yulia Ruzankina, Konstantin Samochernykh, Konstantin Kukanov, Anastasia Nechaeva, Maxim Shevtsov

**Affiliations:** 1 Almazov National Medical Research Centre, Akkuratova Str. 2, 197341 Saint Petersburg, Russia; serge.chudievich@gmail.com (S.C.); pospelovaml@mail.ru (M.P.); ulitinaleks@mail.ru (A.U.); ruzankinajulia@gmail.com (Y.R.); neurobaby12@gmail.com (K.S.); outhall.88@mail.ru (K.K.); nastja-nechaeva00@mail.ru (A.N.); 2 Laboratory of Biomedical Nanotechnologies, Institute of Cytology of the Russian Academy of Sciences (RAS), Tikhoretsky Ave., 4, 194064 St. Petersburg, Russia; 3 Department of Radiation Oncology, Technishe Universität München (TUM), Klinikum rechts der Isar, Ismaninger Str. 22, 81675 Munich, Germany

**Keywords:** high-grade glioma, glioblastoma, laser interstitial thermal therapy, minimally invasive neurosurgery, recurrent glioma, cytoreduction, blood–brain barrier disruption, artificial intelligence

## Abstract

**Background:** High-grade gliomas pose formidable challenges in neuro-oncology, with a median overall survival (OS) of 12–18 months. Laser interstitial thermal therapy (LITT) offers a minimally invasive cytoreductive option for deep-seated or recurrent tumors, achieving ablation rates of 85–98%. In parallel, artificial intelligence and machine learning are increasingly being applied to neuro-oncology to improve diagnosis, treatment planning, and outcome prediction, although these applications remain largely investigational. **Methods:** A literature search was conducted using the PubMed, MEDLINE, Embase, and ClinicalTrials.gov databases. The search terms included “laser interstitial thermotherapy,” “glioblastoma,” and “high-grade glioma”, “machine learning”, “artificial intelligence”. A total of 196 articles were identified. Inclusion criteria comprised primary studies, meta-analyses, and systematic reviews involving human data, with LITT used as a primary or secondary treatment modality. Sixty-nine studies were included in this review, while case reports and animal studies were excluded. **Results:** LITT represents a precision therapy for inoperable gliomas, achieving ablation rates of 85–98%. For primary glioblastoma, median overall survival (mOS) ranges from 11–16 months, and median progression-free survival (mPFS) from 4–9.5 months. In recurrent glioblastoma, LITT demonstrates a median overall survival ranging from 8.5 to 14.1 months and a median progression-free survival of 3–3.5 months with lower complication rates (5.7% vs. 13.8%) and shorter hospital stays (2.2 vs. 7 days). Overall complication rates range from 20–35%, predominantly due to cerebral edema, which is generally responsive to steroid therapy. Its value may be expanded by machine learning tools that integrate clinical, molecular, and imaging features to support patient selection and predict outcomes, though these remain at the proof-of-concept stage. In addition, LITT may serve as a platform for combination therapies, including immunotherapy, chemotherapy, targeted agents, and radiotherapy. **Conclusions:** Based on current evidence, LITT demonstrates outcomes that appear favorable in selected patient populations with high-grade gliomas and may be considered as a treatment option for primary tumors with challenging localization, near-spherical geometry, and volumes of approximately 30 cm^3^. It has a particularly important role in recurrent glioblastomas with similar characteristics, offering efficacy comparable to resection but with an improved safety profile in retrospective comparisons. LITT is evolving from a technically focused ablation method into a data-driven therapeutic platform. Integration with artificial intelligence may improve precision, safety, and personalization, helping define the role of LITT within modern neuro-oncology as higher-quality clinical evidence continues to accumulate.

## 1. Introduction

High-grade gliomas remain one of the most formidable challenges in modern neuro-oncology. The global incidence of high-grade gliomas is approximately 3.2–3.7 per 100,000 population [1,2]. Despite advances in multimodal treatment strategies, including the Stupp protocol (i.e., maximal safe surgical resection followed by radiotherapy with concomitant and adjuvant temozolomide), clinical outcomes remain unsatisfactory. The median overall survival of patients with glioblastoma is approximately 12–18 months, while for grade III gliomas, it ranges from 24–72 months; the five-year survival rate remains as low as 5–10% [3,4,5,6]. These sobering statistics underscore the urgent need for the development and implementation of novel therapeutic approaches to improve patient outcomes.

Among emerging treatment modalities, laser-based technologies in neurosurgery have attracted increasing attention. Broadly, these techniques can be divided into two main categories: laser interstitial thermal therapy (LITT) and photodynamic therapy (PDT). Both approaches utilize laser energy delivered via specialized probes to induce tumor cell destruction, primarily through thermal and photochemical mechanisms.

Laser interstitial thermal therapy is a minimally invasive technique that involves the stereotactic insertion of a laser probe directly into the tumor. This approach is particularly advantageous in cases where lesions are located in deep-seated or functionally eloquent brain regions, making conventional surgical resection high-risk or unfeasible. LITT induces localized hyperthermia, resulting in coagulative necrosis of tumor tissue. In glioblastoma, this method can achieve cytoreduction of approximately 50–70% of tumor volume [7,8]. Additionally, real-time MRI thermography allows for precise monitoring and control of the ablation process, improving both safety and efficacy.

Photodynamic therapy, in contrast, combines laser irradiation with the administration of photosensitizing agents. Upon activation by a specific wavelength of light, these agents generate reactive oxygen species, leading to tumor cell apoptosis and additional cytotoxic effects beyond thermal damage. Unlike LITT, PDT is typically not performed as a stereotactic standalone procedure but rather as an adjunct following surgical resection, aiming to enhance the extent of tumor cell eradication at the resection margins.

Overall, laser-based therapies represent promising and rapidly evolving tools in the treatment of high-grade gliomas. They offer potential advantages in terms of precision, minimal invasiveness, and applicability in otherwise inoperable cases. However, these approaches are not without limitations, including variability in treatment response, technical constraints, and a still-limited body of high-quality clinical evidence. At the same time, neuro-oncology is entering an era of data-driven decision-making. Artificial intelligence and machine learning are increasingly being used to interpret imaging, predict survival, and support individualized treatment planning. These methods are especially promising in LITT, where treatment success depends on multiple interacting variables, including tumor geometry, molecular subtype, trajectory planning, thermal dynamics, and postoperative response.

This review therefore reframes LITT not only as a local ablation technique, but as a platform for precision neuro-oncology. We focus on how AI may improve patient selection, optimize treatment delivery, and guide combination strategies with other therapies.

## 2. Laser Interstitial Thermotherapy

Laser interstitial thermal therapy (LITT) represents an alternative and increasingly important approach to the treatment of gliomas. Although often described as a novel modality, the concept itself is not entirely new. Its origins date back to 1983, when Martin T. Brown first proposed an experimental model for the use of laser-induced thermal energy in the treatment of intracranial lesions [9]. Since then, substantial technological advances, particularly in neuroimaging, stereotactic navigation, and thermal monitoring, have enabled the transition of LITT from an experimental concept to a clinically applicable technique.

Despite these developments, LITT remains, to a significant extent, an emerging and not yet fully established treatment modality. Its adoption in routine clinical practice is still limited, partly due to the relatively small body of high-level evidence and the lack of large randomized controlled trials. In the United States, only a small number of LITT systems have received approval from the U.S. Food and Drug Administration, reflecting the stringent regulatory requirements for medical technologies. Similarly, in Europe and other regions, only a limited number of devices are available for clinical use, often restricted to specialized centers with expertise in stereotactic neurosurgery and intraoperative MRI.

Nevertheless, interest in LITT continues to grow rapidly, driven by its minimally invasive nature, its suitability for treating deep-seated or surgically inaccessible tumors, and its potential to reduce perioperative morbidity. Ongoing technological refinements and accumulating clinical experience are likely to expand its indications and improve its efficacy. As such, LITT occupies an important and evolving niche in the contemporary management of gliomas, bridging the gap between conventional surgery and emerging targeted therapies.

### 2.1. LITT Mechanism of Action

The mechanism underlying LITT is based on the delivery of laser energy, most commonly generated by a neodymium-doped yttrium aluminum garnet (Nd:YAG) laser with a wavelength of 1064 nm [10,11]. This energy induces tumor ablation through several interrelated mechanisms, primarily driven by controlled thermal effects.

The principal mechanism is localized laser-induced hyperthermia, with typical temperatures ranging from approximately 50 °C to 80 °C, which is sufficient to achieve coagulative necrosis of tumor tissue while minimizing damage to the surrounding brain parenchyma [12,13,14]. During this process, emitted photons are absorbed by endogenous chromophores, such as water molecules and hemoglobin, resulting in the conversion of light energy into heat. This thermal energy leads to protein denaturation, disruption of cellular membranes, and ultimately irreversible cell death via coagulation necrosis [15].

Accurate temperature control is a critical component of LITT, as both insufficient and excessive heating may compromise treatment efficacy or safety. Modern LITT systems employ several strategies to achieve precise thermal regulation. Many platforms integrate real-time magnetic resonance imaging (MRI) thermography, allowing for continuous visualization and monitoring of temperature distribution within the target tissue. Other systems incorporate cooling mechanisms, such as circulating CO_2_ or saline within the laser applicator, to prevent overheating and protect adjacent structures.

These technological advances have also led to the introduction of new conceptual frameworks in neuro-oncology. For example, the traditional surgical concept of “extent of resection” is increasingly complemented or even replaced by the concept of the thermal damage threshold, defined as the combination of temperature and duration of exposure required to achieve effective tumor ablation [16,17].

**Figure 1:**
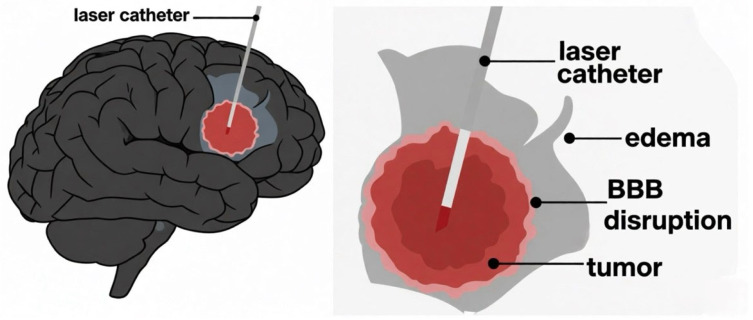
Concept of functional zones in LITT ablation.

Importantly, precise thermal control has revealed that LITT produces several distinct histopathological zones within the treated tissue, each corresponding to different temperature ranges and biological effects (Figure 1; Table 1).

According to the data presented in Table 1, the effects of different temperature ranges in LITT can be utilized in a highly efficient and complementary manner. The central necrotic zone is responsible for achieving direct thermal destruction of tumor cells, enabling maximal volume reduction while preserving surrounding healthy brain tissue. Surrounding this core is the transitional (sublethal) zone, which allows for more delicate cytoreduction at the tumor–brain interface. In addition to partial tumor cell damage, this region is associated with activation of immune responses, including microglial activation, recruitment of CD8+ T lymphocytes, and the release of tumor-associated antigens, thereby helping to counteract the inherently immunosuppressive microenvironment of gliomas.

The outermost region, characterized by transient blood–brain barrier disruption, plays a particularly important role in the peritumoral zone. Here, sublethal hyperthermic exposure induces reversible tissue effects while temporarily increasing blood–brain barrier permeability. This phenomenon is especially advantageous in the context of adjuvant chemotherapy, as it enhances drug delivery to infiltrative tumor margins [18,19,20].

#### 2.1.1. Physical Parameters

The key physical parameters of laser interstitial thermal therapy (LITT) include laser wavelength, power output, optical fiber geometry, and duration of exposure. In modern clinical systems, near-infrared lasers with wavelengths ranging from 980 to 1064 nm are most commonly used, as this range provides an optimal balance between tissue penetration and absorption in brain tissue [21]. The most widely used clinical platforms are the Visualase system (Medtronic; 980 nm diode laser) and the NeuroBlate system (Monteris Medical; 1064 nm Nd:YAG laser) [22].

Shorter wavelengths, such as 980 nm, exhibit slightly higher absorption coefficients in biological tissues, resulting in reduced penetration depth (approximately 8–10 mm) but more rapid heating. In contrast, 1064 nm radiation penetrates deeper into tissue (approximately 12–15 mm) but generally requires higher power to achieve equivalent thermal effects. Experimental systems have also explored wavelengths such as 976 nm. The choice of wavelength significantly influences the morphology of the ablation zone: 1064 nm systems tend to produce more elongated lesions along the fiber axis, whereas 980 nm systems generate more symmetric ablation zones, particularly when diffuser tips are used [20].

The design of laser applicators also plays a crucial role in determining the geometry of the ablation zone. The Visualase system employs a cylindrical diffusing tip (typically 5–10 mm in length), producing an ellipsoidal ablation zone with an approximate axis ratio of 2:1. In contrast, the NeuroBlate system utilizes a directional, side-firing applicator with rotational capability, allowing for the creation of asymmetric ablation patterns tailored to tumor geometry [20]. Controlled rotation of the probe during energy delivery can produce conical or near-spherical ablation zones, although this approach requires advanced trajectory planning and navigation software.

For larger tumors (diameter > 3 cm), multi-probe configurations may be employed, involving up to 4–6 optical fibers. Such approaches are implemented in systems like the TRANBERG Thermal Therapy System, enabling the creation of ablation volumes of up to approximately 5 cm in diameter. However, increasing the number of probes also elevates the risk of intracranial hemorrhage and introduces challenges related to overlapping thermal fields and heat distribution.

In clinical practice, laser power typically ranges from 10 to 15 W, with exposure times between 1 and 5 min. When active fiber cooling is used, treatment duration may be extended to 10–15 min [20]. The biological response of tissue depends on both temperature and duration of exposure. Temperatures of 42–45 °C induce reversible hyperthermic effects, while temperatures of 50–60 °C result in rapid coagulative necrosis. At temperatures exceeding 100 °C, tissue vaporization and carbonization occur, potentially limiting further heat transfer due to the formation of an insulating layer [20].

Thermal dose is often expressed as cumulative equivalent minutes at 43 °C (CEM43). Doses of 10–60 min are associated with irreversible tissue injury, while doses ≥240 min typically result in complete ablation. In clinical settings, the therapeutic target is usually to achieve temperatures of 55–60 °C for 30–60 s, corresponding to an Arrhenius damage integral (Ω) greater than 1 [21].

Heat propagation in biological tissues is determined by their thermophysical properties (i.e., thermal conductivity (k), density (ρ), and specific heat capacity (c)) and by tissue perfusion. Blood perfusion plays a particularly important role through the so-called heat-sink effect, whereby circulating blood dissipates heat and reduces local temperatures. This effect is especially pronounced near large blood vessels (arteries with diameters greater than 1 mm), potentially limiting the extent of effective thermal ablation in these regions [21].

To describe heat transfer in biological tissues, the Pennes bioheat transfer equation is commonly used:

$$\rho c\frac{\partial T}{\partial t}=k\nabla^{2}T+\omega_{b}c_{b}\left(T_{b}-T\right)+Q_{met}+Q_{laser}$$

where *T* is tissue temperature, *k* is thermal conductivity, *ρ* is tissue density, c is specific heat capacity, ω*_b_* is the blood perfusion coefficient, c*_b_* is the heat capacity of blood, *T_b_* is blood temperature (typically 37 °C), *Qmet* represents metabolic heat generation (usually negligible), and *Qlaser* is the volumetric power density of laser irradiation determined by the Beer–Lambert law [21]. This model is widely applied in numerical simulations of LITT and enables the prediction of temperature distribution and ablation volume using finite-element methods [22].

Despite its widespread use, the Pennes model has several limitations. First, it assumes isotropic heat conduction, whereas white matter in the brain is anisotropic; thermal conductivity along axonal tracts may be 30–50% higher than across them, potentially leading to systematic errors in predicting the shape of the ablation zone [23]. Second, perfusion is treated as a constant parameter, although in reality it varies with temperature. At 42–45 °C, vasodilation occurs, increasing perfusion and enhancing the heat-sink effect, while at temperatures above 50 °C, vascular stasis and thrombosis drastically reduce perfusion. Modern models, therefore, incorporate temperature-dependent perfusion functions [21,22,23]. Another limitation is the neglect of phase transitions. At 100 °C, water evaporation absorbs substantial energy (the latent heat of vaporisation of approximately 2260 kJ/kg), temporarily stabilizing temperature. Additionally, carbonisation alters the optical properties of tissue by increasing the absorption coefficient, which is not accounted for in the classical model [21]. Finally, the model assumes tissue homogeneity and does not distinguish between grey and white matter, cystic structures, or calcifications. In reality, ablation zones may deform at tissue boundaries [24]. Nevertheless, the Pennes equation remains the foundation of most commercial simulation platforms, including NeuroBlate and Visualase [4]. To improve accuracy, advanced models incorporating tissue anisotropy and MR angiography data for vessel localization have been proposed [24].

For quantitative assessment of thermal tissue damage, the Arrhenius model is widely used. This model describes protein denaturation as a first-order chemical reaction [22]:

$$\Omega \left(t\right)=\int_{0}^{t}A\cdot exp(-\frac{E_{a}}{RT(\tau )})d\tau$$

where Ω is the thermal damage integral, *A* is the frequency factor, *E_a_* is the activation energy, *R* is the universal gas constant, and *T* is the absolute temperature. When Ω > 1, the probability of cell death exceeds 63%, indicating irreversible thermal injury. This model is widely implemented in modern LITT systems to generate thermal damage maps based on MR thermometry data [23]. However, several limitations should be considered. The parameters *A* and *E_a_* vary significantly between different tissues and proteins. For brain tissue, typical values are *E_a_* ≈ (2.0–2.5) × 10^5^ J/mol and *A* ≈ 10^80^ s^−1^, although these parameters were derived from in vitro experiments and may not fully reflect in vivo conditions. Furthermore, the model does not account for cellular repair mechanisms such as heat shock responses or apoptosis, nor for ischemic necrosis caused by vascular stasis. When heating occurs slowly at lower power levels, reversible tissue changes may occur more frequently than predicted by the model. The threshold value Ω = 1 was empirically derived for mononuclear cells under constant temperature conditions and may require recalibration for variable temperature profiles typical of LITT procedures [24,25]. Despite these limitations, the Arrhenius model remains the gold standard for intraoperative estimation of thermal damage. Some authors suggest using Ω > 3 as a more conservative threshold for complete necrosis [23].

Modern LITT systems incorporate magnetic resonance thermometry, enabling real-time monitoring of tissue temperature [24,25]. The most widely used technique is proton resonance frequency shift thermometry, which relies on the linear dependence of proton resonance frequency on temperature (approximately −0.01 ppm/°C). This method provides temperature measurement accuracy of approximately 1 °C and spatial resolution of 1–2 mm, making it the current standard for monitoring thermal ablation procedures [24,25]. Recent studies have demonstrated that the accuracy and temporal resolution of MR thermometry can be further improved using machine-learning-based reconstruction algorithms and accelerated imaging techniques [25].

Alternative approaches, such as spectral CT thermometry [24], are currently at the experimental stage but may enable thermal ablation procedures to be performed outside MRI-compatible operating suites and are less susceptible to magnetic susceptibility artefacts [24]. The accuracy of CT thermometry (approximately 1.5–2 °C) remains lower than that of MRI; however, this technique represents a promising alternative for patients with contraindications to MRI [25]. A detailed understanding of the biophysical parameters of LITT is directly associated with clinical outcomes.

#### 2.1.2. Future Directions in Biophysical Modeling

The TRANBERG Thermal Therapy System (TwinCool) and other experimental platforms allow for the simultaneous use of up to four laser fibres, enabling ablation volumes of up to 5 cm in diameter. Early clinical studies indicate that the rate of complete tumor ablation can reach 88% for glioblastomas measuring 3–4 cm. However, thermal field interference between multiple probes necessitates numerical optimization: the power delivered to each fiber must be individually modulated to achieve a uniform temperature distribution. Contemporary algorithms based on the superposition method and numerical solutions of the Pennes bioheat equation allow for the calculation of optimal power settings and time delays between probes [25]. Xu et al. (2024) developed a deep neural network for accelerated reconstruction of MR thermography, reducing reconstruction time from approximately 2 s to 50 ms [23]. In parallel, predictive models are being developed to estimate the shape and extent of the necrotic zone preoperatively, based on vascular architecture derived from MR venography and tissue properties [25,26]. Hybrid approaches using physics-informed neural networks integrate the Pennes bioheat equation directly into the neural network architecture, allowing for reconstruction of unmeasured temperature fields and estimation of model uncertainty [25].

Overall, the biophysical parameters of LITT, including laser wavelength, delivered power, fiber geometry, and perfusion-related heat-sink effects, determine not only the size and geometry of the ablation zone but also the risk of complications [21,22,23,24]. The Pennes bioheat equation and the Arrhenius thermal damage model remain the theoretical foundation for intraoperative planning and control; however, their limitations (assumption of isotropic tissue properties, constant perfusion, and neglect of phase transitions) necessitate the development of more advanced computational models and experimental calibration [27]. MR thermometry provides the required temperature measurement accuracy but remains sensitive to motion and susceptibility artifacts [23,24]. Promising directions for further development include: (*i*) multi-probe laser systems with individualized power modulation, (*ii*) hybrid machine learning models for predicting necrosis zones, (*iii*) CT-based thermometry as an alternative for MRI-ineligible patients, and (*iv*) standardization of imaging protocols for ablation zone assessment [24,25]. Addressing these challenges may significantly improve the efficacy and safety of LITT and expand its clinical indications in neuro-oncology.

### 2.2. LITT in Treatment of Grade III Astrocytomas

LITT can be applied in the treatment of various types of gliomas, including IDH-mutant astrocytomas. Moreover, this technique is effective in both primary and recurrent disease settings. Although data on grade III gliomas are more limited compared to glioblastoma, the available evidence is sufficient to evaluate the role of LITT in this subgroup. Most studies report that LITT can achieve ablation of up to 95% of tumor volume in diffuse astrocytomas, with a median extent of ablation ranging from 60% to 100% [27,28,29].

The median duration of the procedure is approximately 210 ± 77.7 min, which is generally shorter than conventional open surgical resection. Postoperative recovery is typically rapid: the median intensive care unit stay ranges from 0 to 2 days, and the overall hospital stay is usually 1–2 days. This accelerated recovery allows for earlier initiation of adjuvant therapies such as chemotherapy and/or radiotherapy [20,30].

In patients with primary grade III gliomas treated with LITT, median progression-free survival (PFS) ranges from 3.5 to 5 years. Three-year PFS rates are reported at 69–75%, while five-year PFS ranges from 42% to 56% [31,32]. These outcomes are strongly influenced by several factors, including the extent of ablation, tumor molecular profile, and the use of adjuvant therapy. In recurrent gliomas, outcomes are more modest: median PFS is approximately 5–6 months, and six-month overall survival reaches 70–80% [33].

With regard to the extent of ablation, a threshold of approximately 95% is generally considered optimal for effective tumor control. However, clinically meaningful results can still be achieved with ablation of at least 85% of tumor volume [34,35].

Postoperative complications associated with LITT are relatively infrequent, occurring in approximately 5–15% of cases [36]. These include both neurological and non-neurological complications. Neurological complications may manifest as cerebral edema, focal neurological deficits, or seizures. Among these, cerebral edema is the most significant and potentially serious complication, occurring in 15–25% of cases [36,37]. Its pathophysiology is multifactorial, involving tumor necrosis, disruption of the blood–brain barrier, and inflammatory or immune-mediated responses. Edema typically peaks within 24–48 h after the procedure and, in 80–90% of cases, responds well to corticosteroid therapy such as dexamethasone [38]. In rare instances, severe edema may lead to mass effect and brain herniation, necessitating decompressive craniectomy.

Focal neurological deficits occur in approximately 10% of patients and commonly include hemiparesis, aphasia, or visual field deficits [39]. These complications are particularly relevant when tumors are located in eloquent brain regions such as the insula, thalamus, or corpus callosum, areas where LITT is often preferred due to the high risk associated with open surgery. In 40–50% of cases, these deficits are transient [40]. However, permanent deficits occur in 4–6% of cases, usually as a result of irreversible thermal injury to surrounding brain tissue. This highlights the importance of precise control of temperature and exposure time during ablation to balance treatment efficacy with safety.

Intracranial hemorrhage is reported in 5–20% of cases and is generally comparable to other stereotactic neurosurgical procedures. Most bleeding events occur along the catheter tract and are typically asymptomatic [41]. However, hemorrhage may also occur within the ablation zone, particularly when a significant portion of tumor tissue remains (>10%). This may be related to residual tumor vascularity or cytokine-mediated vascular fragility [41].

Postoperative seizures occur in approximately 2–5% of patients, a rate comparable to that observed after conventional surgical resection. In high-risk patients, seizures can often be effectively prevented with prophylactic administration of antiepileptic drugs such as levetiracetam [42].

Non-neurological complications are less common, occurring in 0–5% of cases, reflecting the minimally invasive nature of LITT. These include infections and wound-related complications (1–5%), as well as psychiatric symptoms (0–3%). Reported psychiatric manifestations include insomnia, anxiety, and depression, which are often secondary to cerebral edema and typically resolve with its treatment, without the need for specific psychiatric intervention [41].

### 2.3. LITT in Treatment of Newly Diagnosed Glioblastoma

This section focuses specifically on newly diagnosed glioblastoma (IDH-wildtype, WHO grade IV). The biological and clinical characteristics of glioblastoma differ substantially from those of grade III astrocytomas, necessitating separate analyses of clinical outcomes, prognostic factors, and treatment considerations. Glioblastoma remains the most challenging tumor in neuro-oncology, and LITT is increasingly used as a treatment option, particularly when the tumor is located in eloquent brain areas where surgical resection carries a high risk of severe complications (Table 2). In such cases, conventional surgery may not be feasible or advisable, making minimally invasive approaches more attractive. However, LITT remains a relatively restricted modality, with specific selection criteria for appropriate candidates.

Tumor size remains a subject of debate, as no universally accepted protocols currently exist. Most clinical studies suggest that the optimal tumor volume ranges between 30 and 70 cm^3^, with smaller lesions (around 20 cm^3^) being preferable [43,44]. Although this range is broad, it is evident that smaller tumors are more amenable to complete ablation and allow for more precise control of temperature distribution, thereby minimizing damage to surrounding healthy brain tissue. In contrast, larger tumors present significant challenges for LITT. As discussed previously, multiple thermal zones are generated during the procedure, and maintaining adequate control over the ablation process becomes increasingly difficult, even with real-time MRI thermometry [45].

Tumor geometry is another important factor influencing treatment success. Studies indicate that regularly shaped, near-spherical tumors are more suitable for LITT, as they allow for more uniform energy distribution and predictable ablation patterns. In contrast, tumors with irregular geometry are more difficult to treat effectively, as uneven heat distribution may result in incomplete ablation or unintended injury to adjacent structures.

Tumor location also plays a critical role in patient selection. LITT is particularly advantageous for deep-seated glioblastomas located in regions such as the thalamus, insula, basal ganglia, and corpus callosum [46]. These areas are often considered high-risk for open surgical resection due to their functional importance and limited surgical accessibility. While the definition of “unresectable” tumors may vary among neurosurgeons, it is clear that lesions in these locations carry a substantial risk of disabling neurological deficits, which may significantly reduce quality of life and impair the Karnofsky Performance Scale (KPS) score. This, in turn, may preclude patients from receiving further adjuvant therapies such as radiotherapy or chemotherapy. In such scenarios, a safer strategy may involve stereotactic biopsy followed by LITT.

Contraindications for LITT are broadly similar to those for stereotactic biopsy and include a KPS score below 70, significant midline shift (>5 mm), and coagulopathy [35,47]. Careful patient selection is therefore essential to optimize outcomes and minimize risks.

Although LITT shows promise as a treatment for glioblastoma, several important limitations remain. Numerous clinical studies and meta-analyses have been conducted; however, the available data are highly heterogeneous and sometimes contradictory. Reported median overall survival for primary glioblastoma ranges widely from 3.3 to 32.3 months, while progression-free survival varies from 2 to 31.9 months, reflecting considerable variability in study design and patient populations [43]. This heterogeneity limits the strength of evidence-based conclusions.

Several factors contribute to this variability. Many studies are retrospective, non-randomized, and lack control groups. Sample sizes are often small, sometimes including fewer than 30 patients. Furthermore, some studies combine different tumor types within a single cohort, for example, glioblastoma and IDH-mutant grade III/IV astrocytomas, which are biologically and clinically distinct entities and therefore not directly comparable.

Despite these limitations, a general trend can be identified: median overall survival for patients with primary glioblastoma treated with LITT is approximately 11–16 months, with progression-free survival ranging from 4 to 9.5 months [35,44,45,46,47]. Several prognostic factors are associated with improved outcomes, including extent of ablation greater than 90% of the initial tumor volume, MGMT promoter methylation status, smaller initial tumor volume (<20 cm^3^), and a KPS score above 80.

Outcomes are generally poorer in the setting of recurrent glioblastoma. In this group, median overall survival ranges from 3 to 12 months, while progression-free survival is approximately 4–5 months. The six-month progression-free survival rate is reported at 37–59% [44].

Complication rates for LITT in glioblastoma are higher than those observed in grade III astrocytomas, ranging from 20% to 35%, although the overall complication profile remains similar. The most common complication is cerebral edema (15–25%), followed by neurological deficits (10–15%), hydrocephalus (approximately 2%), and seizures (2–5%) [47]. Cerebral edema remains the most clinically significant adverse event. However, it is transient in approximately 80% of cases and can usually be effectively managed with dexamethasone. Neurological deficits such as hemiparesis, aphasia, and other focal impairments are similar in nature to those observed in other gliomas but tend to be permanent more frequently in glioblastoma patients, likely reflecting the more aggressive biology of the disease and the complexity of tumor location.

**Table 2:** LITT for newly diagnosed glioblastoma.

Reference	Number of Patients	OS	PFS	Complications
Ivan et al. (2016) [36]	34	3.3–32.3	2–31.9	Overall complications 5–20%; cerebral edema, new focal deficits, tract hemorrhage
Di et al. (2021) [35]	39	11–16	4–9.5	Edema 15–25%; neurological deficits 10–15%; seizures 2–5%
Muir et al. (2022) [7]	20	11–16	4–9.5	Edema 15–20%, transient/permanent deficits 2–3%, hemorrhage 0–1%
Viozzi et al. (2021) [44]	10	3.3–32.3	2–31.9	Overall complications 20–35%; cerebral edema and neurologic deficit
Viozzi et al. (2023) [45]	10	14.4	4–6	Overall complications 20%: cerebral edema and transient deficits
de Groot et al. (2022) [48]	89	11–16	4–9.5	Overall complications 20–35%; cerebral edema, focal deficits, seizures

### 2.4. LITT in the Treatment of Recurrent Glioblastomas

This section addresses the distinct clinical context of recurrent glioblastoma, in which treatment goals, patient characteristics, and prognostic factors differ substantially from those of newly diagnosed disease. The following discussion should be interpreted in light of the predominantly retrospective, single-center nature of the available evidence. Glioblastoma remains the greatest challenge in modern neuro-oncology, but tumor recurrence represents an even more formidable clinical challenge. Recurrent glioblastoma is often associated with a transition to palliative care, as repeat surgical resection is frequently limited to partial removal or even biopsy alone. At this stage, standard therapies such as temozolomide, bevacizumab, or lomustine tend to lose effectiveness, and the tumor often develops radioresistance [43].

In this context, LITT can play a crucial role as a minimally invasive therapeutic option, offering not only palliation but also a meaningful degree of cytoreduction (Table 3). Patients with recurrent glioblastoma typically present with less favorable clinical characteristics, including older age (median 56–60 years), lower KPS scores (median 60–70), and more pronounced neurological deficits. Additional challenges include prior exposure to radiotherapy and chemotherapy, as well as the presence of pseudoprogression, which can complicate both diagnosis and treatment planning [17,48,49].

Interestingly, the median tumor volume in recurrent glioblastoma is relatively small (approximately 0.9 cm^3^), which contrasts with larger primary tumors and may partially explain why LITT can be effectively applied in this setting [49]. The smaller lesion size allows for more precise thermal control and increases the likelihood of achieving adequate ablation while preserving surrounding brain tissue.

Clinical outcomes following LITT in recurrent glioblastoma remain modest but clinically meaningful. Median overall survival ranges from 3.3 to 8.5 months, while median progression-free survival is approximately 4.3–6.0 months. The six-month progression-free survival rate is reported at 37–59% [17,49,50,51].

Complication rates in this population are approximately 20–25%, with a profile similar to that observed in primary glioblastoma. The most common complication is cerebral edema (7–25%), which is typically responsive to dexamethasone. Neurological deficits (both transient and permanent) occur in approximately 15–20% of patients, while postoperative seizures are reported in around 8% [47].

Overall, LITT appears to be a valuable option in the management of recurrent glioblastoma. It can achieve ablation of approximately 67–90% of tumor volume, providing meaningful cytoreduction. In addition to its direct cytotoxic effects, LITT may also modulate immune responses and induce transient disruption of the blood–brain barrier, potentially enhancing the efficacy of adjuvant chemotherapy [17,49,50,51,52].

However, it is important to emphasize that the current evidence base remains limited. Most available data derive from retrospective cohort studies with relatively small sample sizes and significant heterogeneity. As a result, although LITT is a promising modality, further prospective, randomized controlled trials are necessary to clearly define its role and establish it as a standard treatment option for recurrent glioblastoma.

**Table 3:** LITT in the treatment of recurrent glioblastoma.

Reference	Number of Patients	OS	PFS	Complications
Montemurro et al. (2020) [46]	128	3–12	4–5	Overall 20–25%; cerebral edema 7–25%, transient/permanent neurologic deficits 15–20%, seizures 8%
Muñoz-Casabella et al. (2021) [17]	134	8.5	4.3–6.0	Overall 25%: cerebral edema, deficits, seizures
Nielsen et al. (2026) [50]	30	13.5	-	Overall 20%: cerebral edema and transient deficits
Morello et al. (2024) [53]	13	3.3–8.5	4.3–6.0	Overall 20–25%: cerebral edema, focal deficits, seizures.

LITT is yet to become a considerable choice for the treatment of recurrent glioblastoma but nowadays when we have resection as a gold standard and laser interstitial therapy what are we going to choose in a specific case? Direct comparison between LITT and surgery head-to-head for recurrent glioblastoma reveals an equipoise: survival rates appear comparable (Table 4). Median overall survival rate for LITT is 14.1 months and for resection is 13.8 months from recurrence [47,54]. Total overall survival is about 18 months for both treatment options [42,54]. Progression-free survival following LITT is 3.7 months and for resection is 3.5 months [50,51,52,54]. Crucial part of the efficacy is of course extent of ablation or resection respectively evaluating level of cytoreduction that each method provides. In recurrent glioblastoma median extent of ablation constitutes 67–90%, while median extent of resection is 90–98% [54,55]. These figures, however, should not be interpreted in isolation. In real-world clinical practice, treatment decisions are based on a comprehensive evaluation of tumor characteristics, with location being one of the most critical factors. While surgical resection remains the preferred and often more effective option when gross total or subtotal removal is feasible, its role becomes limited in the case of deep-seated tumors. Lesions located in regions such as the thalamus, limbic system, or corpus callosum, particularly in the setting of recurrent glioblastoma, are often not amenable to safe resection. In such cases, surgery is frequently restricted to stereotactic biopsy without meaningful cytoreduction, making LITT a more suitable therapeutic alternative.

Both LITT and open surgical resection are generally considered safe procedures, but their risk profiles differ. Major neurological complications occur in approximately 5.7% of LITT cases compared to 13.8% in open resection [51,52,54]. The median length of hospitalization is also shorter with LITT, averaging 2.2–2.23 days, whereas patients undergoing craniotomy typically require 3–7 days of inpatient care [42,54]. Hemorrhagic complications are reported in 1–10% of LITT procedures and 5–15% of surgical resections [54,55].

Overall, both approaches demonstrate comparable efficacy and acceptable safety profiles. However, LITT offers advantages in terms of reduced hospital stay, faster recovery, and potentially improved postoperative quality of life. These findings are limited by the retrospective nature of the comparisons and potential selection bias, as patients selected for LITT often present with tumors in less favorable or surgically inaccessible locations.

Given these considerations, it is difficult to define a universally superior treatment modality for recurrent glioblastoma. Instead, the choice between LITT and surgical resection should be guided by principles of personalized medicine. For example, LITT may be the optimal option for small, deep-seated, or otherwise unresectable tumors, whereas open resection may be more appropriate for larger lesions associated with significant mass effect, edema, or midline shift requiring immediate decompression.

### 2.5. Quality of Evidence for LITT Indications

Interpretation of the numerical outcome ranges presented in Section 2.2, Section 2.3 and Section 2.4 requires careful consideration of the substantial between-study heterogeneity, patient selection bias, and confounding by indication. The wide ranges in reported outcomes (e.g., OS 3.3–32.3 months, PFS 2–31.9 months) reflect not only biological variability but also important methodological limitations:

(1) Patient selection bias: studies often include different proportions of patients with favorable versus unfavorable prognostic factors (KPS ≥ 80, MGMT methylation, small tumor volume), affecting reported outcomes;
(2) Tumor location bias: deep-seated tumors (thalamus, basal ganglia) are overrepresented in LITT cohorts compared with surgical series, potentially confounding survival comparisons;
(3) Definition of “unresectability”: there is no consensus definition, leading to variation in the types of tumors included across studies;
(4) Extent of ablation thresholds: studies use different thresholds (70%, 85%, 90%, 95%) for defining adequate ablation, affecting outcome correlations;
(5) Adjuvant treatment heterogeneity: variation in post-LITT chemotherapy, radiotherapy, and immunotherapy regimens significantly impacts reported outcomes;
(6) Publication bias: positive results are more likely to be published, potentially inflating estimates of LITT efficacy;
(7) Small sample sizes: many studies include fewer than 30 patients, limiting statistical power and generalizability.

Table 5 provides a structured summary of the quality of evidence by indication, based on Oxford Centre for Evidence-Based Medicine criteria.

### 2.6. Ongoing Clinical Trials

Modern neuro-oncology relies on a multidisciplinary approach, and exploring combinations of LITT with other treatment modalities represents a promising avenue. However, the current evidence base remains predominantly derived from retrospective studies (Table 6). Currently, the first such trial was underway: the EMITT Trial (NCT05608134, The Netherlands), which randomized patients with unresectable glioblastoma to either biopsy plus LITT or biopsy alone. However, it was unfortunately terminated due to poor recruitment. The published results indicate that, although the trial did not reach its planned enrollment target, the available data provide important preliminary insights into the feasibility and potential effect size of LITT in this challenging patient population. While underpowered to draw definitive conclusions, the trial highlights both the logistical challenges of conducting randomized controlled trials (RCTs) involving LITT and the continued need for high-quality evidence in this field.

Another approach is the combination of LITT with radiotherapy. At present, a pilot study is evaluating LITT followed by hypofractionated radiotherapy (NCT04181684, USA), which is currently recruiting participants.

As previously mentioned, LITT induces transient blood–brain barrier disruption, suggesting that combination with chemotherapy could enhance drug delivery and efficacy. A phase II study investigated LITT followed by six weeks of doxorubicin in 31 patients with recurrent glioblastoma. The results were encouraging: median overall survival improved to 13.6 months, with a six-month overall survival rate of 94% [52]. Progression-free survival was 5.1 months, and six-month progression-free survival was 31% [52].

Additionally, LITT has several immunomodulatory effects, creating a therapeutic window of approximately 30 days that may enhance the efficacy of immunotherapy agents such as vorasidenib, regorafenib, and others [51,53,56,57,58,59]. A phase II study evaluating the combination of LITT with the PD-1 inhibitor pembrolizumab for recurrent glioblastoma reported a median overall survival of 11.4 months and progression-free survival of 10.5 months. Only a single adverse effect was noted, indicating that this combination is both safe and potentially effective [60]. A subsequent detailed follow-up analysis provided deeper insights into the immunological correlates of response, demonstrating that patients with pre-existing T-cell infiltration and post-LITT increases in peripheral CD8+ T-cell clonal expansion derived the greatest benefit [61].

These preliminary studies highlight the potential of LITT as part of multimodal therapy, but larger randomized trials are needed to confirm these benefits and establish standardized protocols for integrating LITT with chemotherapy, radiotherapy, and immunotherapy.

The impact of LITT will be more definitively established once ongoing randomized controlled trials are completed. This technique is likely to be used increasingly in combination with other therapeutic modalities, as LITT appears to enhance the effects of radiation therapy, chemotherapy, and immunotherapy, potentially improving survival outcomes.

The present review synthesizes the evolving role of laser interstitial thermal therapy (LITT) in the management of high-grade gliomas, highlighting its mechanisms, efficacy across tumor grades, and comparative advantages over conventional surgical resection, particularly for deep-seated or surgically challenging lesions. LITT’s zoned thermal effects allow for precise cytoreduction, induce immunogenic cell death, and produce transient blood–brain barrier disruption, making it a compelling alternative to open surgery in anatomically difficult locations.

In grade III astrocytomas, LITT achieves 85–98% tumor ablation, resulting in superior progression-free survival compared with historical controls, supported by relatively short procedure times (median 210 min) and brief hospital stays (1–2 days) [27,28,29,33]. In glioblastoma, outcomes are comparable to Stupp protocol benchmarks: median overall survival for primary tumors is 11–16 months, with median progression-free survival of 4–9.5 months. In recurrent glioblastoma, post-LITT overall survival ranges from 3.3–14.1 months, with six-month progression-free survival of 37–59% when ≥90–95% ablation is achieved [35,44,45,46,47].

Recurrent glioblastoma data highlight LITT’s role as a salvage therapy: despite challenges such as radioresistance and pseudoprogression, ablation rates of 67–90% are attainable, extending overall survival by an average of 8.5 months post-procedure (cumulative survival ~18.6 months from initial diagnosis) [17,49,50,51,52]. In direct comparison with repeat surgical resection, overall survival is similar (14.1 vs. 13.8 months), as is progression-free survival (3.7 vs. 3.5 months), but LITT demonstrates advantages in eloquent locations (thalamus, insula) with substantially fewer permanent deficits (5.7% vs. 13.8%) [52,54,55].

Complication rates remain manageable: 20–35% in glioblastoma and 5–15% in grade III astrocytoma. The most common issues include cerebral edema (15–25%, 80–90% responsive to steroids), neurological deficits (10–15%, with 40–70% transient), and hemorrhage (5–20%, usually along the catheter tract), supporting LITT’s minimally invasive profile relative to open resection [46,55]. Real-time MRI thermometry reduces procedural risk, though heatsink effects from CSF or large vessels necessitate careful multi-fiber planning for irregular or deep lesions exceeding 30–70 cm^3^.

Current limitations of LITT evidence include predominantly retrospective, non-randomized studies with small patient cohorts (n < 30), tumor heterogeneity, and variable ablation thresholds, resulting in wide ranges of reported outcomes (overall survival 3–32 months) and precluding level I evidence. Nevertheless, there is significant potential for ongoing and future randomized controlled trials to establish LITT’s efficacy both as a standalone therapy and in combination with other modalities.

In conclusion, LITT represents a precision cytoreductive strategy for 20–40% of otherwise inoperable gliomas, bridging the gap between palliation and multimodal therapy escalation. Randomized controlled studies are essential to confirm its benefits and refine its personalized role within the management of high-grade gliomas, particularly glioblastomas, where lethality remains high.

## 3. LITT as a Platform for Combination Therapies

LITT should be considered not only as a local cytoreductive technique, but also as a biologically active platform that may enhance the effect of other treatments. Its ability to induce tumor necrosis, disrupt the blood–brain barrier, and trigger local inflammatory responses creates a window of opportunity for multimodal therapy. This is especially relevant in glioblastoma, where local control alone is rarely sufficient and treatment benefit depends on combining surgery-like cytoreduction with systemic disease control.

### 3.1. LITT in Combination with Immune Checkpoint Inhibitors

One of the most promising directions is the combination of LITT with immune checkpoint blockade. Thermal injury caused by LITT induces immunogenic cell death, characterized by microglial activation, recruitment of CD8+ T lymphocytes, and exposure of tumor-associated antigens. These effects may help overcome the immunosuppressive tumor microenvironment characteristic of glioblastoma, where T-cell exclusion and myeloid-derived suppressor cells predominate. In this context, LITT acts as an immune primer, potentially making checkpoint inhibition more effective than monotherapy in untreated tumors. A prospective pilot study (Campian et al., 2021) evaluated LITT followed by standard-dose pembrolizumab (PD-1 inhibitor) in recurrent glioblastoma (n = 10) [60]. The results showed median overall survival of 11.4 months and median progression-free survival of 10.5 months, with only one significant adverse event. This compares favorably to historical pembrolizumab monotherapy benchmarks (mOS 7–9 months in rGBM). The 30-day “immunologic window” post-LITT, driven by transient inflammation and antigen release, likely explains the synergy. From a practical standpoint, immunotherapy is most useful after LITT, when ablation has maximized antigen exposure and created a short-term inflammatory milieu. This post-ablation period (peaking 1–4 weeks post-procedure) is ideal to initiate or intensify immune-based treatment, as blood–brain barrier disruption further facilitates effector cell trafficking. In recurrent glioblastoma, this strategy is especially attractive, where conventional options (bevacizumab, lomustine) are limited and immune responsiveness is poor [51,58,59]. Ongoing trials, such as those combining LITT with vorasidenib or regorafenib, are exploring this window to potentiate immune modulation. Preclinical data reinforce the concept: sublethal hyperthermia upregulates MHC-I expression and PD-L1 on tumor cells, sensitizing them to PD-1/PD-L1 blockade. A 2024 meta-analysis (Sharma et al.) of LITT-immunotherapy combinations in recurrent brain tumors reported improved PFS-6 (37–59%) versus LITT alone, though larger RCTs are needed [51]. In summary, LITT combined with checkpoint inhibitors leverages ablation-induced immunogenicity to address glioblastoma’s immune “cold” state. With mOS/PFS gains in early studies, this combination warrants prioritization in phase III trials like EMITT extensions (NCT05608134).

### 3.2. LITT in Combination with Chemotherapy

LITT may significantly improve chemotherapy delivery by transiently disrupting the blood–brain barrier. Local hyperthermy induces reversible BBB opening lasting up to 1 month, confirmed by dynamic contrast-enhanced MRI in preclinical rat models and human studies. This hyperthermia-mediated effect increases local permeability, allowing for superior penetration of systemic drugs into the peritumoral region and residual infiltrative margins. Phase II clinical data provide concrete evidence. A single-arm study (Butt et al., 2021; n = 27) combined LITT with doxorubicin (40 mg/m^2^ for 6 weeks) in recurrent glioblastoma, reporting mOS 13.6 months, 6-month OS 94%, and PFS-6 31%—outcomes exceeding historical LITT monotherapy benchmarks [57]. Doxorubicin, normally BBB-limited due to P-glycoprotein efflux, achieved enhanced peritumoral delivery during the post-LITT window, as evidenced by gadolinium extravasation on follow-up imaging. This directly validates LITT as a local delivery enhancer beyond cytoreduction. The effect is particularly relevant for adjuvant treatment after ablation. BBB disruption peaks at days 3–14 post-LITT and persists 1–4 weeks, creating an optimal window to administer agents with poor brain penetration (doxorubicin, lomustine, topotecan) [53]. Preclinical data show 2–5-fold increased drug accumulation in peritumoral tissue during this period. This strategy is especially useful for second-line agents: doxorubicin, carboplatin. Ongoing trials (NCT04181684) explore LITT combined with hypofractionated RT/chemotherapy, exploiting both BBB opening and rapid post-LITT recovery (hospital stay 2.2 vs. 7 days post-resection). In summary, LITT transforms from pure ablation to a chemo-sensitizing platform for adjuvant chemotherapy. With demonstrated survival gains and mechanistic validation, LITT combined with chemotherapy warrants prioritization in future trials, particularly for rGBM where systemic options are exhausted.

### 3.3. LITT Combined with Radiotherapy

Radiotherapy remains a cornerstone of high-grade glioma management, and LITT complements it through multiple synergistic mechanisms. First, hyperthermic cytoreduction reduces viable tumor burden, improving target volume definition and potentially sparing adjacent normal tissue from radiation dose. Second, LITT induces thermal radiosensitization by impairing DNA repair, BBB disruption and effects persisting 1–4 weeks post-ablation. Third, LITT’s rapid recovery profile (median hospital stay 2.2 days vs. 7 days post-resection) enables earlier radiotherapy initiation (within 7–14 days), avoiding delays that compromise survival in frail patients. Prospective pilot data support feasibility. NCT04181684 evaluates MR-guided LITT followed by hypofractionated RT (35 Gy/5 fractions) in recurrent high-grade gliomas post-standard therapy. Early safety data show no radiation-related toxicities, with median time-to-radiotherapy of 10.5 days. Preclinical models confirm synergy: LITT combined with radiotherapy increases apoptosis and reduces regrowth vs. radiotherapy alone, attributed to microvascular normalization and enhanced oxygen diffusion post-ablation. Hypofractionated regimens match LITT recovery kinetics better than conventional fractionation. Ongoing trials (EMITT NCT05608134) and preclinical synergy data position LITT combined with radiotherapy as a viable platform for rGBM, where options dwindle. With lower morbidity than re-resection this combination warrants phase III validation.

## 4. Advanced Imaging and AI Integration

### 4.1. Treatment Planning

**Figure 2:**
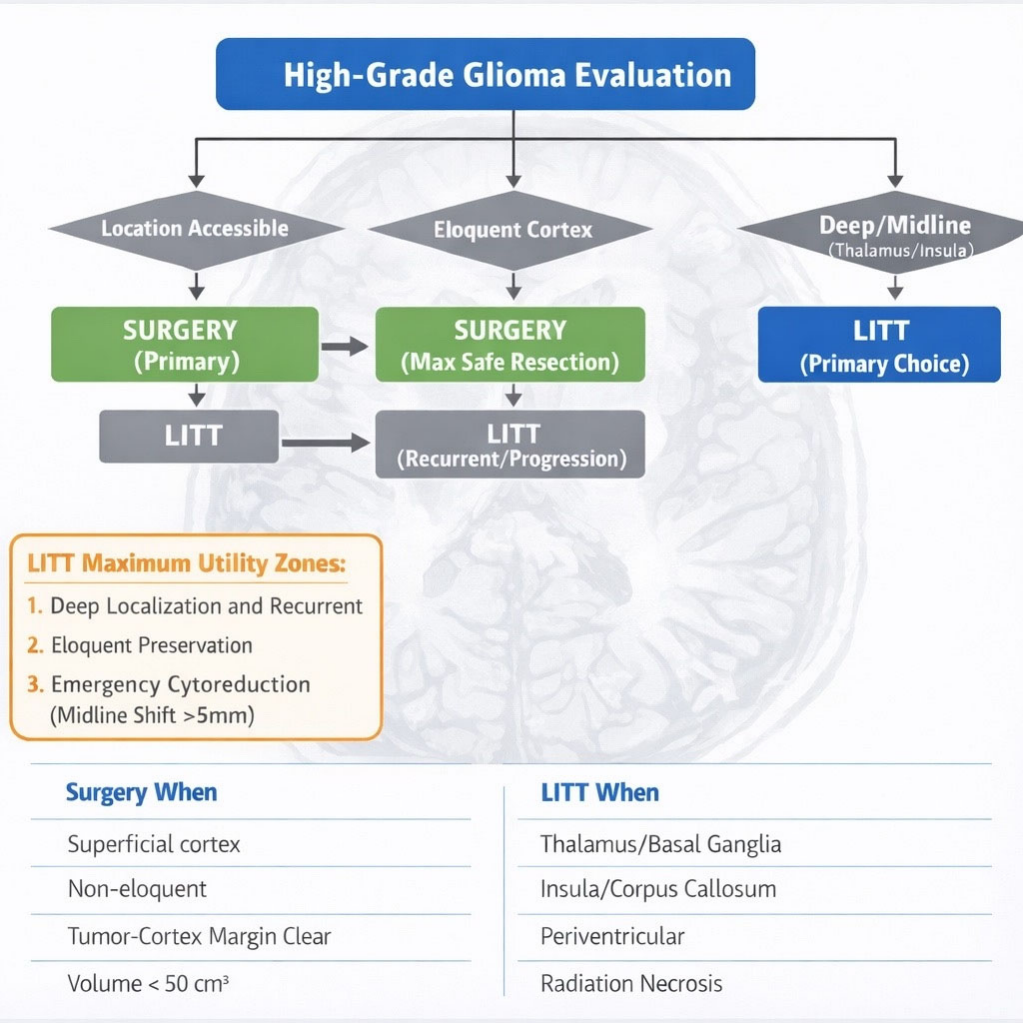
AI-based decision making conceptualization.

Laser interstitial thermal therapy generates extensive imaging and procedural datasets, which has prompted investigation into artificial intelligence applications for this modality. However, it is important to emphasize that most AI applications described in this section remain at the proof-of-concept or early feasibility stage, with prospective validation in multicenter cohorts required before clinical implementation (Figure 2). Nevertheless, real-time MR thermometry, as the current standard for intraoperative guidance, remains constrained by several technical limitations. These are the following: motion artifacts from respiration or bulk flow, magnetic susceptibility distortions, and the heat-sink phenomenon adjacent to high-flow vessels collectively compromise spatial and temporal fidelity. Moreover, phase lags between measured temperature elevation and actual coagulative necrosis hinder precise intraoperative demarcation of the ablation isotherm. In irregularly shaped or periventricular gliomas, these factors contribute to underestimation of the necrotic core by 15–25% and unpredictable perilesional edema exceeding 30% of cases [62]. One of the possible solutions is the use of perioperative radiomics. Preoperative radiomics transforms routine multiparametric MRI into high-dimensional feature sets that inform treatment planning with favorable granularity. Volumetric shape descriptors, first-order intensity statistics, gray-level co-occurrence matrices capturing texture heterogeneity, and lesion-to-eloquent structure spatial gradients collectively predict technical feasibility and safety. Machine learning classifiers, typically convolutional neural networks or gradient-boosted trees, trained on 200–500 cases achieve 88–94% accuracy in identifying optimal probe trajectories, defined as >90% target coverage while respecting 2 mm safety margins to corticospinal tracts or language networks [61,63]. In practice, these models excel for multifocal lesions requiring dual-probe configurations or tumors abutting the ependyma, where manual planning fails 30–40% of attempts. Beyond trajectory selection, radiomic signatures forecast procedure-specific risks: peritumoral edema, tract hemorrhage and permanent neurological deficit. Multicenter implementation could reduce inter-operator variability, establishing LITT as a reproducible intervention comparable to stereotactic radiosurgery [64].

### 4.2. Outcome Prediction and Perioperative Risk Stratification

Preoperative risk stratification synthesizes radiomic, clinical, and molecular inputs through ensemble algorithms or deep transformer architectures trained on multi-institutional LITT cohorts. Feature engineering emphasizes ablation, peritumoral relative cerebral blood volume, and T1/T2 MRI mismatch volume. Duke’s convolutional neural networks model validates preoperative 12-month OS prediction [63]. Postoperative surveillance elevates this paradigm further, employing recurrent networks to scrutinize serial multiparametric MRI sequences. These models detect progression signals, such as accelerating FLAIR volume expansion beyond 20% or rising delta-relative cerebral volume, in a median 21 days before radiologist consensus with 92% sensitivity. Such foresight empowers different adaptive strategies for the treatment of each single patient. Ultimately, prognostic modeling may improve the clarity of outcome prediction for LITT, potentially transforming it from a salvage maneuver into a more prognostically informed platform. However, these models require prospective validation before they can be considered clinically reliable, and their performance in multicenter settings remains to be established.

### 4.3. Digital Twin Concept

Patient-specific digital twins are fusing biophysical fidelity with artificial intelligence to simulate ablation dynamics on an individualized basis. These virtual replicas begin with finite-element discretization of the Pennes bioheat equation, augmented by anisotropic conductivity tensors derived from diffusion tensor imaging accounting for white matter tract thermal gradients and overlaid with vascular scaffolds from MRI. Physics-informed neural networks propel this framework forward by embedding conservation principles: mass, momentum and laser energy. Validated with intraoperative thermometry, physics-informed neural networks reconstruct occult thermal fields with mean absolute error of 1.2 °C, while forecasting Arrhenius damage contours with 92% volumetric fidelity [65]. In preliminary feasibility studies, preoperative trajectory optimization has shown potential for 18–27% ablation volume expansion [66]. These findings require confirmation in larger, prospective studies before clinical implementation.

It should be emphasized that while digital twin technology holds promise, these systems are currently at an early stage of development. The cited studies represent proof-of-concept investigations with small sample sizes and single-center designs. Prospective validation, standardization of modeling approaches, and assessment of clinical utility in multicenter cohorts are essential next steps.

### 4.4. AI-Assisted Decision Making Framework

Precision treatment selection of effective LITT implementation may be enhanced by artificial intelligence which bridges the gap between anatomical feasibility and oncologic benefit. The following framework is presented as a conceptual proposal based on published feasibility studies and expert opinion. It has not been clinically validated and should not be interpreted as a clinically implemented algorithm. The proposed decision algorithm integrates preoperative multiparametric MRI radiomics, molecular profiling, and digital twin simulation into a unified workflow that stratifies patients across three critical dimensions: technical feasibility, expected survival benefit, and complication risk. Initial processing extracts over 1000 quantitative features from standard imaging sequences such as tumor sphericity, peritumoral relative cerebral blood volume elevation and minimum distance to corticospinal and other tracts. Convolutional neural network ensembles, validated on multi-institutional cohorts exceeding 350 patients, deliver technical feasibility scores with 88–94% accuracy, defining lesions amenable to single-probe ablation achieving ≥ 90% target coverage [61,63]. Parallel digital twin simulation merging anisotropic Pennes bioheat modeling against patient-specific vascular maps forecasts ablation isotherms across competing trajectories, routinely identifying power modulation strategies that expand necrotic core volume by 18–27%. Risk stratification then coalesces these outputs into low-risk (predicted progression-free survival > 9 months) versus high-risk cohorts (mPFS < 4 months) [61,63,64,65,66]. For deep-seated or recurrent glioblastomas in the thalamus, insula, or periventricular regions where resection risks are high, LITT emerges as the preferred cytoreductive platform when AI feasibility is powerful enough. Postoperative surveillance employs recurrent neural networks monitoring serial imaging, detecting progression signals 21 days ahead of radiologic consensus with 92% sensitivity. This computational continuum, spanning preoperative optimization through adaptive postoperative management, represents a conceptual vision for integrating LITT within modern neuro-oncology’s precision paradigm. Prospective validation studies are needed to determine whether these computational approaches can translate into meaningful improvements in clinical outcomes.

## 5. Conclusions

In conclusion, LITT represents a precision cytoreductive approach that may provide a valuable therapeutic option for a subset of patients with otherwise challenging-to-resect gliomas. Current evidence suggests that LITT may be particularly suitable for tumors with unfavorable surgical accessibility, relatively spherical geometry, and moderate volumes (approximately ≤ 30 cm^3^), where achieving adequate cytoreduction with conventional surgery carries substantial risk. Its role appears especially relevant in recurrent glioblastoma, where LITT may offer disease control outcomes comparable to those reported for surgical resection in selected patients while potentially providing a more favorable safety profile according to retrospective analyses.

However, interpretation of these findings requires caution, as the current evidence base is predominantly derived from retrospective, single-center, and heterogeneous studies, with considerable variability in patient selection, treatment protocols, and outcome assessment. Therefore, the use of LITT should currently be considered within a personalized treatment-selection framework rather than as a broadly established standard of care. The identification of appropriate candidates remains a critical challenge and requires integration of tumor biology, anatomical characteristics, patient-specific risk factors, and available therapeutic alternatives.

Prospective multicenter studies and randomized controlled trials are needed to define the true clinical benefit of LITT, establish comparative effectiveness relative to surgical and nonsurgical approaches, and clarify its optimal integration into multimodal treatment strategies for high-grade gliomas, particularly glioblastoma, where therapeutic resistance and mortality remain major challenges. Future advances in artificial intelligence may further enhance the implementation of LITT by supporting individualized patient selection, preoperative planning, treatment optimization, and outcome prediction. However, these AI-driven applications remain largely investigational and require prospective validation before routine clinical adoption.

## Figures and Tables

**Table 1:** LITT thermal zones.

Thermal Zone	Temperature (°C)	Mechanism	Zone of Interest
Central necrotic zone	50–100	Immediate cell death induced by hyperthermia and protein denaturation	Tumor cells
Transitional sublethal zone	41–45	Autoimmune reaction, internal apoptosis	Tumor cells and peritumoral zone
Blood–brain barrier disruption zone	38–42	Reversible blood–brain barrier disruption up to 1 month	Peritumoral zone

**Table 4:** LITT and resection for recurrent glioblastoma.

Reference	N (LITT)/N (Resection)	mOS LITT/mOS Resection	mPFS LITT/mPFS Resection	Complications
Fadel et al. (2022) [54]	30/30	14.1/13.8	3.7/3.5	5.7%/13.8%
Barnett et al. (2016) [42]	8/22	10–14 both	3–5 both	1–10%/5–15%

**Table 5:** Quality of evidence for LITT by indication. * Oxford Centre for Evidence-Based Medicine Levels: I (systematic review of RCTs), II (individual RCT), III (non-randomized controlled studies), IV (case series), V (expert opinion/bench studies).

Indication	Evidence Level *	Study Types	Sample Sizes	Key Limitations
Newly diagnosed GBM	III	Retrospective cohort, single-center	*n* = 10–89	Selection bias, lack of controls, heterogeneity
Recurrent GBM	III	Retrospective cohort, single-center	*n* = 13–128	Selection bias, confounding by indication
Grade III astrocytoma	III	Retrospective cohort, single-center	*n* = 12–45	Small samples, mixed IDH status
LITT vs. resection	III	Retrospective matched cohorts	*n* = 8–30 (LITT) vs. 22–30 (resection)	Non-randomized, selection bias
Combination therapies	IIb-III	Phase I/II trials, retrospective	*n* = 10–31	Small samples, lack of controls
AI-assisted planning	V	Proof-of-concept, feasibility studies	*n* = 20–500	Not validated, experimental

**Table 6:** Ongoing trials for LITT.

Trial	Condition	Design and Phase	Scheme	Key Endpoints and Features
EMITT trial (NCT05608134, The Netherlands)	Newly diagnosed, primary irresectable glioblastoma	Multicenter randomized controlled trial, phase III	Arm 1: stereotactic biopsy followed by MR-guided LITT and standard radio-chemotherapy Arm 2: stereotactic biopsy alone with standard radio-chemotherapy	First large RCT designed to test whether adding LITT to biopsy improves outcomes in unresectable glioblastoma
Hypofractionated radiotherapy after LITT (NCT04181684, USA)	Recurrent high-grade gliomas	Prospective pilot/phase I–II	MR-guided LITT followed by hypofractionated radiotherapy	Safety of sequential LITT and hypofractionated radiotherapy
LITT + doxorubicin in recurrent glioblastoma	Recurrent glioblastoma after standard treatment	Single-arm phase II	MR-guided LITT followed by systemic doxorubicin	mOS ≈ 13.6 months; 6-month OS 94%, mPFS 5.1 months, 6 months PFS 31%
LITT + pembrolizumab (PD-1 inhibitor) in recurrent glioblastoma	Recurrent glioblastoma	Prospective pilot, phase II	MR-guided LITT followed by pembrolizumab	mOS 11.4 months, mPFS 10.5 months.

## Data Availability

No new data were created or analyzed in this study.

## Abbreviations

The following abbreviations are used in this manuscript:

LITT	Laser interstitial thermotherapy
BBB	Blood–brain barrier
mOS	Median overall survival
mPFS	Median progression-free survival
PFS	Progression-free survival
CT	Computer tomography
MRI	Magnetic resonance imaging
IDH	Isocitric Dehydrogenase
MGMT	Methylated-DNA–protein-cysteine methyltransferase
GBM	Glioblastoma
rGBM	Recurrent glioblastoma
